# Willingness to pay for a dengue vaccine in Iran: Insights from a contingent valuation study

**DOI:** 10.1371/journal.pntd.0013869

**Published:** 2026-01-02

**Authors:** Moslem Soofi, Ali Kazemi-Karyani, Zahra Alipoor, Hekmatolla Mahmoodi, Behzad Karamimatin

**Affiliations:** 1 Social Development and Health Promotion Research Center, Health Policy and Promotion Institute, Kermanshah University of Medical Sciences, Kermanshah, Iran; 2 Department of Clinical Psychology, school of medicine, Kermanshah University of Medical Sciences, Kermanshah, Iran; 3 Research Center for Environmental Determinants of Health, Health Institute, Kermanshah University of Medical Sciences, Kermanshah, Iran; Wadsworth Center, UNITED STATES OF AMERICA

## Abstract

**Background:**

Vaccination is crucial for controlling infectious diseases like dengue. Vaccine acceptance and willingness to pay (WTP) significantly impact vaccination programs’ success but remain unexplored in Iran. This study estimates the population’s WTP for a dengue vaccine (DV) and its determinants.

**Methodology and principal findings:**

This descriptive-analytical study involved 1,031 adults aged over 18 years. WTP for the DV was estimated using the double-bounded dichotomous choice (DBDC) contingent valuation method (CVM) with an interval data approach. Model parameters were estimated via the maximum likelihood method. Data analysis was conducted using Stata version 17. Approximately 66% of participants indicated a WTP for a DV. The mean WTP was 11,129,000 Iranian Rials (IRR) (95% CI: 10,374–11,884; p < 0.001) (≈13.9 USD; 1 USD ≈ 800,000 IRR, December 2024–May 2025). Compared with reference groups, being married, having high school or university education, middle or high socioeconomic status, and moderate or high perceived dengue risk were associated with higher WTP, while participants aged ≥61 years had lower WTP.

**Conclusions:**

Higher risk perception, greater socioeconomic status, higher level of educational, and older age were identified as significant determinants of WTP for the DV. Subsidizing the vaccine for lower-income groups and raising awareness about dengue infection risk, particularly among individuals with lower educational levels, may enhance vaccine uptake and contribute to more effective dengue prevention efforts in Iran.

## Author summary

Dengue fever is an illness spread by mosquitoes that can lead to serious health problems. Vaccination is one way to prevent dengue, but in Iran, we do not know how much people are willing to pay for such a vaccine or what influences their decision. In our study, we asked more than 1,000 Iranian adults about their WTP for a DV. About two-thirds said they would be willing to pay, with an average amount of around 14 US dollars. People were more likely to pay if they were older, had higher education and income, or felt that dengue was a greater risk. These results suggest that if dengue becomes a health concern in Iran, reducing the vaccine cost for lower-income groups and raising public awareness about dengue could increase vaccine uptake. Our findings provide useful evidence for health authorities to plan future dengue prevention strategies and make vaccines more accessible to everyone.

## Introduction

Dengue fever is a rapidly spreading vector-borne viral disease caused by the dengue virus and primarily transmitted by *Aedes* mosquitoes, particularly *Aedes aegypti.* It represents a major global public health concern, resulting in substantial economic and resource burden on health services in endemic settings [1]. Globally, dengue affects approximately 390 million people annually, with around 96 million developing clinical symptoms ranging from mild fever to severe, potentially fatal conditions such as dengue hemorrhagic fever and dengue shock syndrome. The World Health Organization (WHO) estimates that over 4 billion people across more than 110 countries are at risk, underscoring the disease’s extensive global impact [2–4]. In recent years, the incidence of dengue has risen markedly, driven by factors such as urbanization, climate change, and increased human mobility, which have expanded mosquito habitats and extended the geographic distribution of the virus [5].

Dengue fever has recently emerged as a public health concern in Iran due to the establishment of *Aedes aegypti* in southern regions and the occurrence of dengue outbreaks in neighboring countries. This emerging threat highlights the urgent need for effective prevention strategies [6]. Currently, vector control remains the primary method for dengue prevention; however, it faces challenges such as insecticide resistance and environmental concerns [7]. Vaccination presents a promising strategy for controlling dengue transmission. In 2023, the World Health Organization (WHO) recommended the widespread use of vaccine in regions with high dengue incidence [5,8]. Nevertheless, the success of vaccination programs depends largely on public acceptance and WTP, which significantly influence vaccine uptake and the overall effectiveness of prevention efforts [9,10].

WTP is an economic measure that reflects the monetary value individuals assign to a health intervention, capturing their preferences and perceived benefits. It provides essential insights to policymakers regarding resource prioritization, subsidy allocation, and pricing, particularly in resource-constrained settings. The CVM is a widely utilized survey technique for estimating WTP for non-market goods, such as vaccines, by presenting hypothetical scenarios and eliciting respondents’ maximum WTP. Estimating the public’s understanding of the economic benefits of a given vaccine (DV) and the value they place on it is crucial for conducting cost-benefit analyses, guiding resource allocation decisions, and formulating strategies related to vaccine inclusion in public health programs or private markets, as well as pricing policies [11,12].

Multiple factors influence WTP for vaccines, including sociodemographic variables, socioeconomic status, as well as health-related factors. Understanding these determinants is crucial for designing targeted communication and equitable vaccination programs [10,13–15].

Despite the increasing risk of dengue in Iran, there is a paucity of research addressing the population’s WTP for DV in this context. To date, the potential acceptability of the DV and the population’s WTP have not been assessed. Most existing studies focus on endemic regions in Southeast Asia and Latin America [14–16], which differ epidemiologically and socioeconomically from Iran. Therefore, context-specific evidence is needed to guide national vaccination policies effectively.

This study aims to estimate the general population’s WTP for the DV in Iran using the CVM and to identify the factors influencing WTP. The findings will offer valuable insights for policymakers in designing effective, equitable, and sustainable dengue vaccination programs, thereby enhancing Iran’s preparedness in addressing this emerging public health threat.

## Methods

### Ethics statement

This study was approved by the Ethics Committee of Kermanshah University of Medical Sciences. (Ethics code: IR.KUMS.REC.1403.343). The study was conducted in accordance with the ethical principles outlined in the Declaration of Helsinki. A written informed consent was obtained from all participants in this study.

### Study design and sample

This study employed a cross-sectional survey design to estimate the general population’s WTP for the DV in Iran. Data were collected between December 2024 and May 2025. The single population proportion formula was employed to calculate the sample size,


$$N=\frac{Z_{\text{1}-\frac{\sigma }{\text{2}}}^{\text{2}}P(\text{1}-P)}{d^{\text{2}}}$$

Where N is the determined sample size, Z is the value of the standard normal distribution at a 95% confidence level (1.96), P is the assumed proportion of individuals exhibiting the characteristic of interest. Since there was no established estimate for the proportion of individuals willing to pay, a value of 50% was used for sample size calculation. d represents the acceptable margin of sampling error (0.04) [17]. Considering a 10% non-response rate and to minimize sampling error, the final sample size was increased to N = 1031. A convenience sampling method was employed in this study.

### Survey instrument

The survey instrument was developed by a multidisciplinary team of experts, including two health economists, three healthcare management specialists, two health promotion experts, one psychologist, one expert in health and social welfare, one health policy expert, and one social worker. The questionnaire was primarily designed to elicit WTP using a series of DBDC questions. It also collected information on sociodemographic characteristics, perceived risk of dengue infection, history of relevant illnesses, and self-rated overall health. Overall health was measured on a 10-point scale and categorized into three groups for analysis: fair (1–4), good (5–6), and very good (7–10). Sociodemographic variables included sex, age, marital status, educational level, and socioeconomic status (SES). Marital status was categorized as married or single. Owing to the small proportions of widowed (0.9%) and divorced (0.85%) participants, these groups were merged with the single category to ensure adequate cell sizes, and the combined category (“single/divorced/widowed”) was used as the reference group in the analysis. Educational level was categorized as secondary and lower, high school, or university degree; participants with no formal education (1.94%) or only primary education (3.59%) were combined with the secondary-education category to maintain sufficient cell sizes and statistical reliability. SES was self-reported on a 10-point scale considering income, occupation, education, and place of residence (1 = very low, 10 = very high) and grouped for analysis into low (1–4), middle (5–6), and high (7–10). The validity of the questionnaire was established through qualitative evaluation by experts’ opinion. Furthermore, a pilot survey involving 100 participants was conducted to identify ambiguous or problematic items, evaluate the appropriateness of bid values, and ensure the questionnaire was clear, understandable, and culturally appropriate across diverse subgroups of age, education, and socioeconomic status. Given the complexity of contingent valuation questions, this larger pilot helped detect and address potential comprehension issues and inconsistencies before full-scale administration

### Econometric estimation and eliciting WTP

To elicit WTP, the CVM was employed using a DBDC format. This approach enables more efficient utilization of respondents’ information and is regarded as appropriate due to its superior statistical efficiency in estimating parameter variance, thereby producing more precise confidence intervals [18].

Participants were initially asked whether they would be willing to pay a specified initial price for the DV. The initial bid (Bid_I_) values were derived from a pre-survey conducted with 100 individuals who provided their WTP for the vaccine using an open-ended format. To mitigate starting point bias, four distinct initial bid values were used. The value of initial bid was randomly assigned to each participant as recommended in the literature. If the respondent answered “no” to the initial bid, a lower bid (bid_L_) was subsequently offered in the follow-up bid; conversely, if the respondent answered “yes,” a higher bid (bid_H_) was presented. Specifically, a “yes” response to the follow-up bid resulted in doubling the initial price, while a “no” response led to halving it [19].

Consequently, each participant provided two responses corresponding to these bid intervals. Based on the pattern of responses to the DBDC questions, WTP estimates can be categorized into four possible intervals, reflecting the range within which the true WTP lies: [12,18,20].

(1) “yes-yes”, where: Bid_H_ ≤ WTP < ∞
(2) “yes-no”, where: Bid_I_ ≤ WTP < Bid_H_
(3) “no-yes”, where: Bid_L_ ≤ WTP < Bid_H_, and
(4) “no-no”, where: 0 < WTP ≤ Bid_L_

The econometric estimation assumes that WTP can be modeled as follow:


$${\text{WTP}}_{\text{i}}\;\;({\text{z}}_{\text{i}},\;{\text{u}}_{\text{i}})\;=\;{\text{z}}_{\text{i}}^{\prime }\beta \;+{\text{u}}_{\text{i}}\;\;\text{and}\;\;{\text{u}}_{\text{i}}\;\;\approx \;\;\text{N}\;(\text{0},\sigma^{\text{2}}\;)$$

where z_i_ is a vector of explanatory variables, β is a vector of parameters and u_i_ is an error term.

The following function were maximized to estimate the parameters of the model:


$$\begin{array}{c} \begin{array}{c} \sum_{i=\text{1}}^{N}[d_{i}^{yn}\text{ln}\left(\Phi \left(z_{i}^{\prime }\frac{\beta }{\sigma }-\frac{{Bid}_{L}}{\sigma }\right)-\Phi \left(z_{i}^{\prime }\frac{\beta }{\sigma }-\frac{{Bid}_{H}}{\sigma }\right)\right)+d_{i}^{yy}\text{ln}\left(\Phi \left(z_{i}^{\prime }\frac{\beta }{\sigma }-\frac{{Bid}_{H}}{\sigma }\right)\right)\\ \;+\;d_{i}^{ny}\text{ln}\left(\Phi \left(z_{i}^{\prime }\frac{\beta }{\sigma }-\frac{{Bid}_{L}}{\sigma }\right)-\Phi \left(z_{i}^{\prime }\frac{\beta }{\sigma }-\frac{{Bid}_{H}}{\sigma }\right)\right)+\;d_{i}^{nn}\text{ln}\left(\text{1}-\Phi \left(z_{i}^{\prime }\frac{\beta }{\sigma }-\frac{{Bid}_{L}}{\sigma }\right)\right)] \end{array} \end{array}$$

Furthermore, $d_{i}^{yn}$, $d_{i}^{yy}$, $d_{i}^{ny}$, and $d_{i}^{nn}$ are indicator variables that take the value of one or zero, depending on the relevant case for each individual, implying that a given individual contributes the likelihood function’s logarithm in only one of its four parts. β and σ were estimated using the maximum likelihood method [21,22]. WTP estimates were converted to US dollars using an average exchange rate of 1 USD = 800,000 IRR, based on the monthly mean exchange rates from the Iranian free market between December 2024 and May 2025.

## Results

The mean age of respondents was 35.85 (SD ± 11.44). More than half (52.28%) of the respondents were women, 60.43% had a university degree, 48.69% belonged to the age group of 31–56 and most of the participants (62.46) were married. 84.19% of respondents stated that they had previous vaccination experience, and 55.87% of participants had a low level of risk perception. (Table 1)

**Table 1:** Characteristics of the participants, cross-sectional survey, 2025.

Variables		N (%)
Sex	Male	492 (47.72)
	Female	539 (52.28)
Age group	18–30 years	384 (33.75)
	31–45 years	502 (48.69)
	46–60 years	142 (13.77)
	61 years or more	39 (3.78)
Marital status	Married	644 (62.46)
	Single/Widowed/Divorced	387 (37.54)
Education level	Secondary and lower	121 (11.74)
	High school	287 (27.84)
	University degree	623 (60.43)
Socioeconomic status	Low	207 (20.08)
	Middle	319 (30.94)
	High	505 (48.98)
Self-rated general health	Poor/ Fair	27 (2.62)
	Good	129 (12.51)
	Very good	875 (84.87)
Level of risk perception	Low	576 (55.87)
	Moderate	302 (29.29)
	High	153 (14.84)
Having Previous vaccination experience	Yes	163 (15.81)
	No	868 (84.19)

Note: 1 US$ ≈ 800,000 IRR at the time of the study.

**Fig 1:**
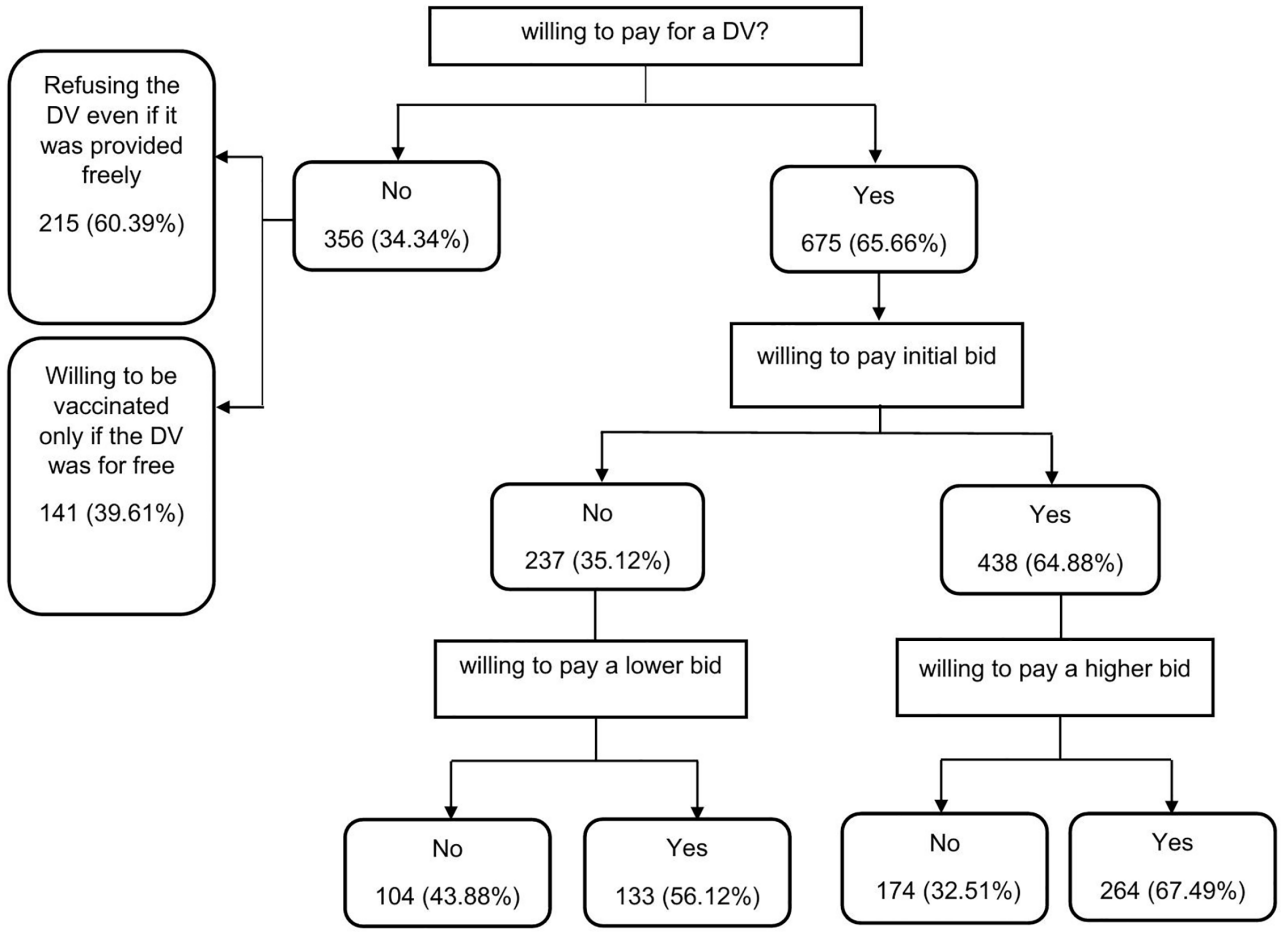
Summary statistics of responses to the double-bounded dichotomous choice questions.

Of the sample, 675 (65.47%) participants were willing to pay for a DV. The remaining 356 (34.53%) individuals stated that they were unwilling to pay for a DV, of which 215 (60.39%) individuals stated that they would refuse the DV even if it was given to them for free, and 141 (39.60%) individuals stated they would only be vaccinated if the vaccine was free. In addition, 264 (25.61%) of participants stated that they would pay both the initial bid and the higher second bid, while 174 (16.88%) participants stated that they would pay the initial bid but not the higher second bid (Fig 1).

Table 2 indicates the results from the DBDC models and the WTP. Individuals with higher education and those in middle or high socioeconomic groups show substantially greater WTP compared with their respective reference categories. A clear gradient also emerges for risk perception, as both moderate and high levels of perceived risk show strong and statistically significant positive associations with WTP. Being married is associated with higher WTP, whereas participants aged 61 years or older have significantly lower WTP. Sex, prior vaccination experience, and self-rated health do not show statistically significant associations. Overall, these findings highlight socioeconomic, educational, and perceptual factors as key determinants of WTP in this sample.

**Table 2:** The effect of explanatory variables on the WTP of individuals for DV.

Variables		Coefficient	standard error	z	P-value
Sex (Ref. Female)	Male	17.02533	74.18069	0.23	0.818
Age group (Ref. 18–30 years)	31–**45** years	70.91413	92.35199	0.77	0.443
	46–60 years	37.42484	125.9721	0.30	0.766
	61 years or more	-596.0903	213.7574	-2.79	0.005
Marital status (Ref. Single/Widowed/Divorced)	Married	185.7165	86.94156	2.14	0.033
Level of education (Ref. Secondary and lower)	High school	389.2098	143.0429	2.72	0.007
	University degree	291.8941	133.3404	2.19	0.029
Socioeconomic status (Ref. Low)	Middle	330.8415	112.2774	2.95	0.003
	High	449.8573	105.8353	4.25	0.000
Self-rated general health (Ref. Poor/ Fair)	Good	348.0991	260.8133	1.33	0.182
	Very good	446.7328	244.9895	1.82	0.068
Level of risk perception (Ref. Low)	Moderate	289.1804	82.55358	3.50	0.000
	High	924.9947	108.7269	8.51	0.000
Having Previous vaccination experience (Ref. No)	Yes	13.39954	98.64062	0.14	0.892
-cons		-314.27	286.35	-1.10	0.272
Sigma					
_cons		826.35	37.783	21.87	0.000

Sample: 675, Log likelihood = -866.75, Wald chi2 [14] = 133.39, Prob > chi2 = 0.0000.

Table 3 shows the estimated mean WTP for the basic and expanded model. The basic model is based on the assumption that there are no control variables. The expanded model depicted the best-fit model when control variables were taken into account. The results indicated that the mean of the WTP for DV for the basic and expanded model was 11623.80 (Thousand Rials) (CI 95%: 10817.80 - 12429.70) and 11129.20 (Thousand Rials) (CI 95%: 10374.20 - 11884.20), respectively. The estimated WTP was statistically significant in both models.

**Table 3:** Estimation of double-bounded discrete choice models and WTP estimates for the basic and expanded model, 2025.

	Mean WTP (IRR Thousands)	Standard. Error	P-value	Confidence interval 95%
Basic model (no covariates)	11623.8 (14.5 USD)	411.1	< 0.001	10817.80 -12429.70
Expanded model (with covariates)	11129.2 (13.9 USD)	385.1	< 0.001	10374.20 -11884.20

## Discussion

We employed the CVM using a DBDC format to estimate the WTP for a hypothetical DV and to examine its associated factors in Iran. To the best of our knowledge, this is the first study to estimate WTP for a DV in Iran using this approach. The findings revealed that approximately 66% of participants were willing to pay for a hypothetical DV, indicating a moderate level of acceptance when compared to other dengue-endemic countries.

For instance, Yeo and Shafie (2018) [15] reported a higher acceptance rate of 88.4% in Penang, Malaysia, while Kabir et al. (2021) [16] observed a comparable rate of 71.2% in Bangladesh for at least one of three hypothetical vaccine scenarios. In Indonesia [23], acceptance rates also varied considerably, as Hadisoemarto and Castro (2013) reported an acceptance rate of 94.2%. However, it is important to note that high acceptance does not always correspond to high WTP, as illustrated by the relatively low median WTP observed in their study [23].

These variations in acceptance and WTP are likely attributable to differences in dengue epidemiology, cultural perceptions of vaccination, public health messaging, and socioeconomic conditions across countries [10,24,25].

Regarding the mean WTP, this study estimated an average of approximately 14 USD for the DV in Iran, based on an exchange rate of 1 USD ≈ 800,000 IRR during the study (December 2024–May 2025). This amount is higher than the mean WTP reported by Harapan et al. [26] (4 USD) and Hadisoemarto and C. Castro [23] (1.4 USD) in Indonesia, and also somewhat higher than the per-dose WTP of 9.45 USD found in Malaysia [15]. Conversely, it is lower than WTP values observed in Bangladesh, where the mean WTP ranged from 15.70 USD to 29.70 USD per dose, depending on the vaccine’s effectiveness [16]. Additionally, Lee et al. (2015), in a multi-country study, reported mean WTP values of 24.46 USD in Vietnam, 47.26 USD in Thailand, and 30.45 USD in Colombia (25) [24], all of which exceed the estimate found in our study. Other studies also reported higher mean WTP, including 67.4 USD in Vietnam [27] and 27–32 USD in the Philippines [28].

The variation in WTP across studies likely reflects differences in socioeconomic status, perceived risk of dengue, disease prevalence and incidence, vaccine characteristics, and factors related to national health systems [10]. Furthermore, each study employed distinct hypothetical DV scenarios within their contingent valuation frameworks and utilized different statistical models to estimate WTP [15].

Consistent with findings from Vietnam [Vo et al., 2018] [14], Malaysia [Yeo and Shafie, 2018] [15], and the multi-country study [Lee et al., 2015] [25], our study identified higher educational level and higher socioeconomic status as significant positive determinants of WTP, suggesting that financial capacity and health literacy play central roles in shaping vaccine valuation. This broadly suggests that individuals with greater access to information, higher health literacy, and more financial capacity are more inclined to invest in preventive health measures like vaccination. Moreover, the observed link between lower educational level and reduced WTP highlights the need for targeted public health interventions. Vaccination campaigns should specifically focus on individuals with lower education levels, as they may have limited health literacy and awareness of dengue risks. Tailoring communication strategies to this demographic is essential to bridge informational gaps, enhance understanding of vaccine benefits, and ultimately boost acceptance and uptake among those less inclined to seek vaccination [10]. The positive association between socioeconomic status and WTP for a DV strongly supports subsidizing vaccination costs for lower-income groups. This policy could significantly increase vaccine uptake, aligning with public health goals, while higher-income individuals are capable of paying the full price. This approach ensures equitable access by removing financial barriers for vulnerable populations [26].

We also found that individuals with a higher risk perception were more likely to pay for a DV. The positive relationship between increased risk perception and WTP for a DV aligns with the understanding that a heightened awareness of a disease’s severity and personal susceptibility drives a greater willingness to invest in protection [13,20,29]. This underscores the importance of effective risk communication strategies in shaping vaccine demand.

Yeo and Shafie (2018) in Malaysia highlighted that respondents with higher dengue knowledge score were more likely to accept the vaccine. While not directly about risk perception, knowledge often informs perceived risk. They also stated that the WTP amount reflected the value people placed on avoiding risks related to dengue fever. Specific groups of people, particularly those who felt more vulnerable and perceived a higher need to protect themselves placed higher value for the vaccine. This directly links perceived vulnerability/risk to WTP [15]. Given its modifiable nature, the level of risk perception warrants significant attention in public health policy. Since it’s a key determinant of vaccine acceptance, well-designed interventions to raise individuals’ perceived risk for dengue fever are essential to foster a positive attitude toward vaccination. This underscores the crucial need to improve public awareness regarding both dengue fever and the importance of vaccination [26,30,31].

It’s worth noting that this study was conducted when dengue fever was not yet epidemic in Iran. It is anticipated that an epidemic scenario would likely elevate the population’s risk perception, potentially leading to even higher levels of WTP for a vaccine. This suggests that public health strategies for vaccine introduction should consider the dynamic nature of risk perception, adapting communication efforts as disease prevalence changes.

The study found that married individuals exhibited a higher WTP for the DV. This finding aligns with the results of Vo et al., who identified marital status as a significant factor influencing WTP [14]. The higher WTP among married respondents may be attributed to an increased sense of responsibility for family health and well-being, as married individuals often prioritize the protection of their household members.

We found that individuals aged 61 years or older had a significantly lower WTP for the DV. Similarly, Nguyen et al. reported that older adults were less inclined to pay for the DV [27]. The reduced WTP observed among the elderly in the Iranian context may be attributable to several factors, including lower perceived benefits, or greater reliance on public health financing, the presence of comorbid conditions, lower income levels during retirement, or a perceived limited life expectancy to benefit from long-term vaccine protection. This age-related disparity in WTP merits further investigation to elucidate the underlying behavioral and economic factors. In line with previous studies [28,32]. Sex was not significantly associated with WTP for the DV in our study. Although women made up a slightly larger share of the sample (52.3%), sex was not a significant predictor of WTP in the regression model. One explanation is that both genders reported comparable levels of risk perception and similar past vaccination experiences, which may reduce gender differences in payment preferences. The absence of a gender effect suggests that vaccine financing policies may not need to differentiate by sex, and that more influential determinants—such as socioeconomic status, education, and perceived risk—should guide intervention design.

This study has several limitations that should be considered when interpreting the findings. First, the use of convenience sampling may introduce sampling bias and limit the generalizability of the results, as the sample may not fully reflect the demographic and socioeconomic diversity of the Iranian population. Therefore, caution is warranted when extrapolating these findings. Future studies should consider probability-based sampling methods to improve representativeness and external validity. Second, the study was conducted under a hypothetical DV scenario at a time when dengue fever had not yet reached epidemic levels in Iran. This context may have influenced respondents’ WTP, as the perceived urgency and relevance of vaccination may differ significantly during an actual outbreak. Consequently, the applicability of these findings in a future epidemic context may vary.

Despite these limitations, we assert that this study has effectively captured essential baseline data on the WTP for a DV in Iran. This foundational information offers valuable insights into public acceptance and economic valuation of the vaccine, serving as a critical basis for future research and informing policy development.

In conclusion, this study estimated a statistically significant mean WTP for a DV in Iran, calculated at 11,129.2 thousand IRR (≈14 USD). Higher socioeconomic status, greater perceived risk of contracting dengue fever, and higher educational attainment were positively associated with increased WTP. These findings underscore the critical importance of targeted public health education to enhance risk awareness, alongside tailored subsidy programs aimed at supporting lower-income and less-educated populations, which together can improve vaccine uptake and strengthen dengue prevention efforts in Iran.
